# Coupling of phagocytic NADPH oxidase activity and mitochondrial superoxide production

**DOI:** 10.3389/fcvm.2022.942736

**Published:** 2022-07-28

**Authors:** Sergey I. Dikalov, Anna E. Dikalova, Igor A. Kirilyuk

**Affiliations:** ^1^Vanderbilt University Medical Center, Nashville, TN, United States; ^2^N.N. Vorozhtsov Novosibirsk Institute of Organic Chemistry SB RAS, Novosibirsk, Russia

**Keywords:** superoxide, mitochondria, phagocytic NADPH oxidase, electron paramagnetic resonance (EPR), spin probe

## Abstract

Superoxide radical plays an important role in redox cell signaling and physiological processes; however, overproduction of superoxide or insufficient activity of antioxidants leads to oxidative stress and contributes to the development of pathological conditions such as endothelial dysfunction and hypertension. Meanwhile, the studies of superoxide in biological systems represent unique challenges associated with short lifetime of superoxide, insufficient reactivity of the superoxide probes, and lack of site-specific detection of superoxide. In this work we have developed ^15^N-and deuterium-enriched spin probe ^15^N-CAT1H for high sensitivity and site-specific detection of extracellular superoxide. We have tested simultaneous tracking of extracellular superoxide by ^15^N-CAT1H and intramitochondrial superoxide by conventional ^14^N-containing spin probe mitoTEMPO-H in immune cells isolated from spleen, splenocytes, under basal conditions or stimulated with inflammatory cytokines IL-17A and TNFα, NADPH oxidase activator PMA, or treated with inhibitors of mitochondrial complex I rotenone or complex III antimycin A. ^15^N-CAT1H provides two-fold increase in sensitivity and improves detection since EPR spectrum of ^15^N-CAT1 nitroxide does not overlap with biological radicals. Furthermore, concurrent use of cell impermeable ^15^N-CAT1H and mitochondria-targeted ^14^N-mitoTEMPO-H allows simultaneous detection of extracellular and mitochondrial superoxide. Analysis of IL-17A- and TNFα-induced superoxide showed parallel increase in ^15^N-CAT1 and ^14^N-mitoTEMPO signals suggesting coupling between phagocytic NADPH oxidase and mitochondria. The interplay between mitochondrial superoxide production and activity of phagocytic NADPH oxidase was further investigated in splenocytes isolated from Sham and angiotensin II infused C57Bl/6J and Nox2KO mice. Angiotensin II infusion in wild-type mice increased the extracellular basal splenocyte superoxide which was further enhanced by complex III inhibitor antimycin A, mitochondrial uncoupling agent CCCP and NADPH oxidase activator PMA. Nox2 depletion attenuated angiotensin II mediated stimulation and inhibited both extracellular and mitochondrial PMA-induced superoxide production. These data indicate that splenocytes isolated from hypertensive angiotensin II-infused mice are “primed” for enhanced superoxide production from both phagocytic NADPH oxidase and mitochondria. Our data demonstrate that novel ^15^N-CAT1H provides high sensitivity superoxide measurements and combination with mitoTEMPO-H allows independent and simultaneous detection of extracellular and mitochondrial superoxide. We suggest that this new approach can be used to study the site-specific superoxide production and analysis of important sources of oxidative stress in cardiovascular conditions.

## Introduction

Reactive oxygen species such as superoxide (O_2_^•^) play an important role as second messengers in cell signaling in physiological regulation of cellular differentiation, proliferation, motility, and immune responses (1). Meanwhile, increased superoxide production or impairment of antioxidant system leads to excessive superoxide levels and oxidative injury (2). Increased superoxide production, therefore, is not always associated with oxidative stress and indiscriminative targeting of superoxide may result in off-target suppression of cellular functions. To study the specific pathophysiological role of superoxide we need to define the sources of superoxide and employ site-specific detection of superoxide radicals.

There are several key sources of superoxide including NADPH oxidase, mitochondria, and xanthine oxidase (3). The analysis of superoxide sources is further complicated by expression of various NADPH oxidase isoforms (i.e., Nox1, Nox2, Nox4 etc.) and several superoxide producing mitochondrial sites with distinct regulations (1). It is important to note that different cell types express distinct sources of superoxide and, therefore, detection methods must be tailored to specific cells. Inflammatory cells represent a critical source of superoxide not only in inflammatory conditions but also in metabolic conditions, cardiovascular disease, hypertension, and end-organ damage (1). Activation of immune cells is associated with increased superoxide production and leads to release of pro-inflammatory cytokines such as IL-17A and TNFα which contribute to development of pathological conditions (4). Understanding the redox regulation of immune cells activity can allow a better therapeutic targeting of immune system reducing the inflammatory injury.

**Figure 1:**
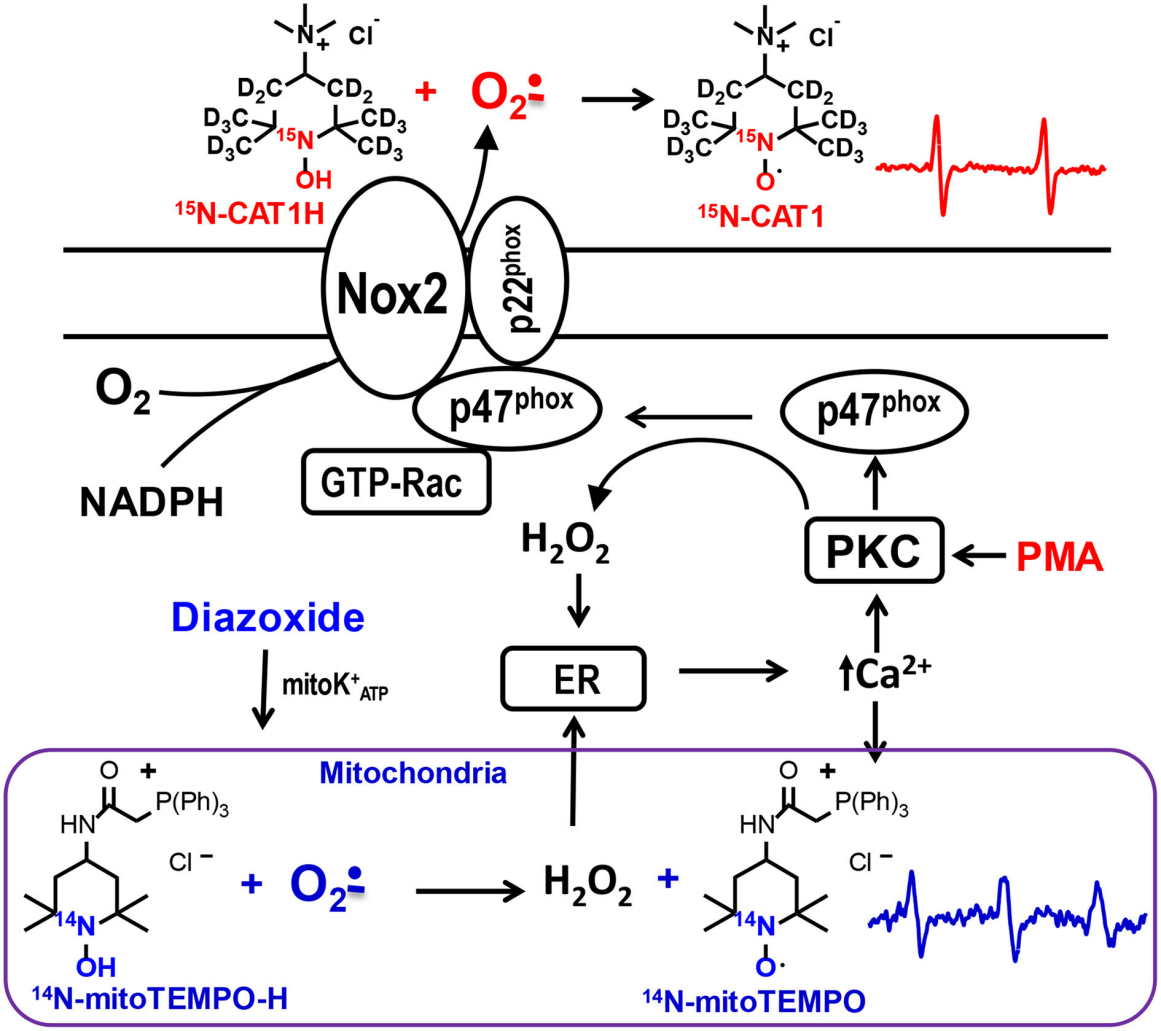
Schematic presentation of site-specific localization of ^15^N-CAT1H and ^14^N-mitoTEMPO-H spin probes allowing separate detection of extracellular and mitochondrial superoxide production by phagocytic NADPH oxidase and mitochondria in splenocytes. ^15^N-CAT1H and ^14^N-mitoTEMPO-H are added to splenocytes at 500 μM and 25 μM correspondingly which results in detection of extracellular superoxide predominantly by cell impermeable ^15^N-CAT1H due to its excess by 20-fold compared with ^14^N-mitoTEMPO-H. Meanwhile, cell permeable and mitochondria targeted ^14^N-mitoTEMPO-H accumulates in mitochondrial matrix where it is capable of superoxide detection producing ^14^N-mitoTEMPO nitroxide.

Cells isolated from spleen include a variety of immune cells such as T and B lymphocytes, dendritic cells, and macrophages (5). Splenocytes represent a convenient model to study immune cells. Superoxide in splenocytes is mainly produced by phagocytic NADPH oxidase and mitochondria (Figure 1) (6). Despite numerous studies, analysis of superoxide in these cells represents unique challenges associated with short lifetime of superoxide, insufficient reactivity of the superoxide probes, and lack of site-specific detection of superoxide. We have previously described detection of extracellular superoxide using cell-impermeable positively charged spin probe CAT1H (7). and analysis of mitochondrial superoxide using cell-permeable mitochondria-targeted lipophilic cation spin probe mitoTEMPO-H (7). We proposed that combination of ^15^N-CAT1H and ^14^N-mitoTEMPO-H can provide simultaneous detection of extracellular and mitochondrial superoxide due to site-specific localization of spin probes and non-overlapping spectra of ^15^N-CAT1 and ^14^N-mitoTEMPO nitroxide products (Figure 1). In this work we have used ^15^N-and deuterium-enriched spin probe ^15^N-CAT1H for high sensitivity and site-specific detection of extracellular superoxide. We have tested simultaneous detection of extracellular superoxide by ^15^N-CAT1H probe and mitochondrial superoxide by ^14^N-mitoTEMPO-H probe in splenocytes under basal or stimulated conditions by inflammatory cytokines IL-17A and TNFα, NADPH oxidase activation by PKC agonist PMA (8), or treated with of mitochondrial inhibitors rotenone and antimycin A using Electron Paramagnetic Resonance (EPR). Interestingly, EPR analysis showed parallel increases in ^15^N-CAT1H and ^14^N-mitoTEMPO signals suggesting coupling between phagocytic NADPH oxidase and mitochondria. Stimulation of phagocytic NADPH oxidase led to increase in both extracellular and mitochondrial superoxide, while Nox2 depletion in splenocytes attenuated angiotensin II mediated stimulation and inhibited both extracellular and mitochondrial PMA-induced superoxide production. We suggest that this method can provide a new insight in redox regulations of immune cells.

## Materials and methods

### Reagents

Xanthine oxidase was purchased from Roche Diagnostics GmbH (Manheim, Germany). Nitroxide radicals TEMPOL and mitoTEMPO were purchased from Enzo Life Sciences (San Diego, USA). All other reagents were obtained from Sigma (St Louis, MO). Preparation of mitoTEMPO-H, ^14^N-CAT1H and ^15^N-CAT1H is described below.

**Scheme 1:**
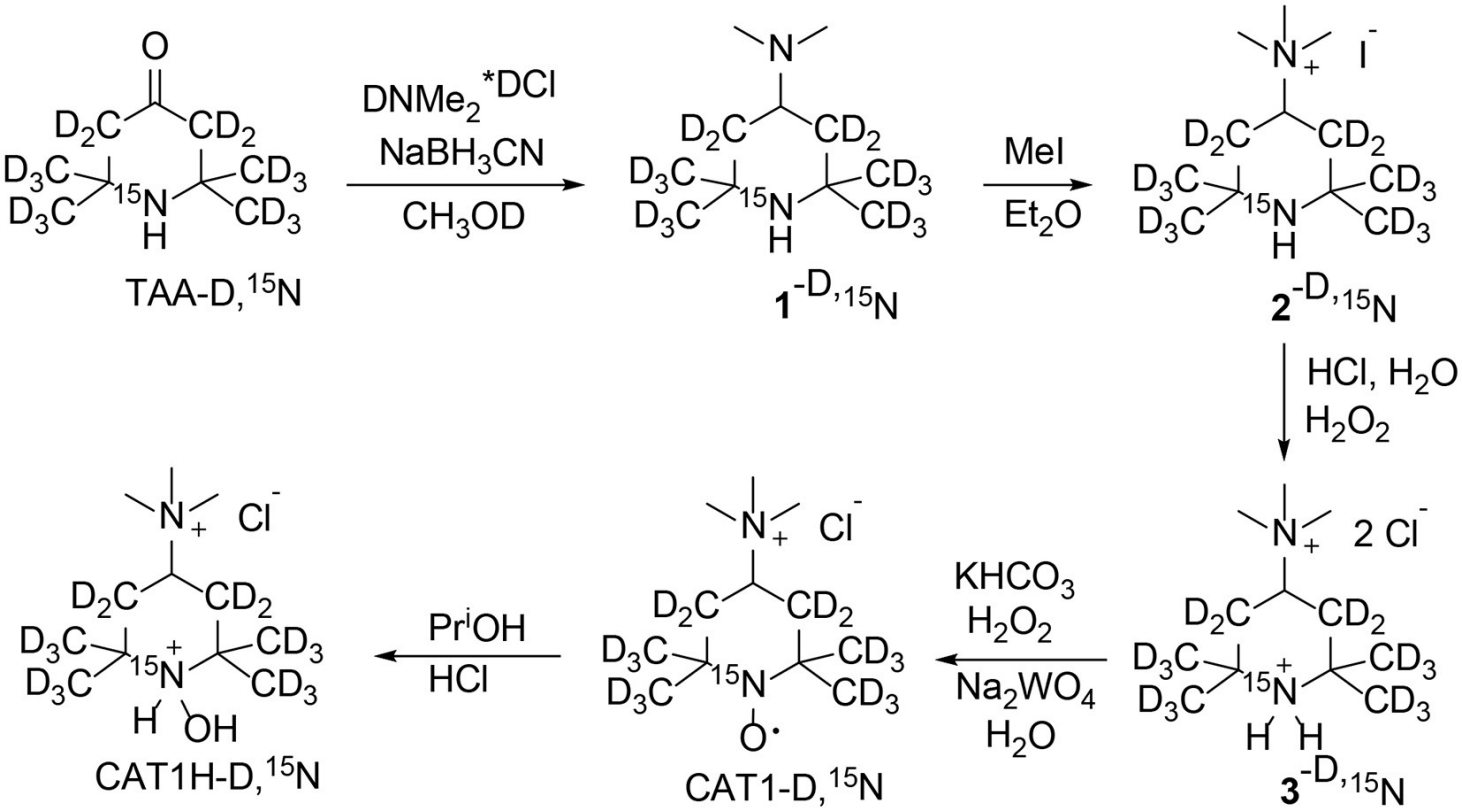
Synthesis of 1-hydroxy-2,2,6,6-tetra(^2^H_3_)methyl-4-trimethylammonio(3,3,5,5-^2^H_4_,1-^15^N) piperidine chloride (CAT1H-D,^15^N).

*1-Hydroxy-2,2,6,6-tetra(*^2^*H*_3_*)methyl-4-trimethylammonio (3,3,5,5-*^2^*H*_4_*,1-*^15^*N)piperidine chloride hydrochloride* was prepared from 4-oxo-2,2,6,6-tetra(^2^H_3_)methyl(3,3,5,5-^2^H_4_,1-^15^N) piperidine (TAA-D,^15^N) (9) according to Scheme 1 using literature procedures (10) with some modifications. In brief, TAA-D,^15^N was subjected to reductive amination with sodium borohydride in the presence of excess of dimethylammonium hydrochloride. Careful alkylation of the resulting 4-dimethylamino-2,2,6, 6-tetramethylpiperidine (**1**) with iodomethane in analogy to literature (10) afforded corresponding quaternary salt **2**, iodide. Here we suggest a simple procedure to replace iodide to chloride without ion-exchange chromatography. The aqueous solution of **2** was acidified with excess of hydrochloric acid and iodide was oxidized with hydrogen peroxide to elemental iodine. The precipitate of crystalline iodine was filtered off and the remaining traces of iodine were removed via extraction with benzene. Evaporation of the colorless solution afforded hydrochloride of quaternary amine **3**, chloride. The resulting hydrochloride was neutralized to free base and oxidized in analogy to literature (10), to give CAT1. The nitroxide CAT1 was reduced to corresponding hydroxylamine CAT1H with a mixture of ethanol and 2-propanol in hydrochloric acid in analogy to literature (11). Each procedure was tried with conventional (^14^N, ^1^H) compounds prior to application to isotope-enriched material.

*4-Dimethylamino-2,2,6,6-tetramethylpiperidine (****1****)*. A mixture of triacetonamine (1 g, 6.45 mmol), dimethylamine hydrochloride (2 g, 24.5 mmol), sodium cyanoborohydride (0.5 g, 7.96 mmol), and methanol was stirred at room temperature for 48 h. The inorganic precipitate was filtered off, methanol was distilled off in vacuum. Saturated solution of NaCl was added to the residue, the mixture was basified with NaOH (0.5 g) and extracted with diethyl ether (3 ×10 mL). The extract was dried with Na_2_CO_3_ and ether was distilled off in vacuum to give **1** (1.08 g, 91%), colorless liquid, IR (neat), ν_max_ cm^−1^: 2955, 2931, 2862, 2820, 2771, 1456, 1375, 1365, 1325, 1242, 1219, 1164, 1103, 1030, 856; ^1^H NMR (300 MHz, CDCl_3_, δ): 0.78 (2H t, J 12 Hz), 0.93 (6H s), 0.99 (6H s), 1.53 (2H dd, J_1_ 12 Hz, J_2_ 3 Hz), 2.07 (6H s), 2.44 (1H tt, J_1_ 12 Hz, J_2_ 3 Hz); ^13^C{^1^H} NMR (75 MHz, CDCl_3_, δ): 28.26 and 34.95 (Me), 40.65 (CH_2_), 40.84 (NMe), 50.58 (CMe_2_), 55.84 (CH); cf (10).

*4-Dimethylamino-2,2,6,6-tetra(*^2^*H*_3_*)methyl(3,3,5,5-*^2^*H*_4_*,1-*^15^*N)piperidine (*1-D,^15^N*)* was prepared similarly from TAA-N, ^15^D (9) in CH_3_OD, dimethylamine hydrochloride was enriched with deuterium via dissolving in D_2_O and evaporation in vacuum. IR (neat), ν_max_cm^−1^: 2931, 2862, 2818, 2771, 2212, 2123, 2069, 1470, 1456, 1441, 1267, 1209, 1196, 1169, 1093, 1053, 993, 856, 654, 613; ^1^H NMR (300 MHz, CDCl_3_, δ): 2.11 (6H s), 2.46 (1H m); ^13^C{^1^H} NMR (75 MHz, CDCl_3_, δ): 27.36 (m, CD_3_) and 34.00 (m, CD_3_), 40.2 (m, CD_2_), 40.95 (NMe), 50.14 (m, CMe_2_), 55.71 (m, CH).

*2,2,6,6-Tetramethyl-4-trimethylammoniopiperidine iodide (****2****)* was prepared using the procedure described in literature (10). Methyl iodide (0.8 g, 5.6 mmol) was added at once to a solution of **1** (1.06 g, 5.76 mmol) in diethyl ether (15 mL). The mixture was allowed to stand at room temperature for 24 h. The precipitate was filtered and washed with diethyl ether to give **2** monohydrate (1.4 g, 72%), colorless crystals, mp 216–217°C (*i*-PrOH – MeOH). Elemental analysis, found: C, 41.88; H, 8.23; N, 8.14; I, 36.57; calcd. for C_12_H_29_N_2_OI: C, 41.86; H, 8.49; N, 8.14, I, 36.86 %. IR (KBr), ν_max_ cm^−1^: 3508, 3354, 3296, 3248, 3198, 2971, 2870, 1645, 1492, 1481, 1390, 1371, 1340, 1261, 1230, 1194, 972, 885, 866, 804; ^1^H NMR (300 MHz, CDCl_3_ – CD_3_OD, δ): 0.99 (6H s), 1.00 (6H s), 1.24 (2H t, J 12 Hz), 1.80 (2H dd, J_1_ 12 Hz, J_2_ 3 Hz), 2.91 (9H s), 3.73 (1H tt, J_1_ 12 Hz, J_2_ 3 Hz); ^13^C{^1^H} NMR (75 MHz, CDCl_3_ – CD_3_OD, δ): 27.03 and 33.06 (Me), 36.78 (CH_2_), 50.73, 50.76 and 50.79 (NMe), 51.60 (CMe_2_), 69.06 (CH); cf (10).

*2,2,6,6-Tetra(*^2^*H*_3_*)methyl-4-trimethylammonio(3,3,5,5-*^2^*H*_4_*, 1-*^15^*N)piperidine iodide (***2**-D,^15^N*)* was prepared similarly from 1-D,^15^N. IR (KBr), ν_max_ cm^−1^: 3506, 3354, 3239, 3194, 3012, 2946, 2224, 2125, 2071, 1645, 1491, 1479, 1417, 1241, 1200, 1161, 958, 879; ^1^H NMR (300 MHz, CDCl_3_ – CD_3_OD, δ): 2.89 (9H s), 3.79 (1H m); ^13^C{^1^H} NMR (75 MHz, CDCl_3_ – CD_3_OD, δ): 26.54 (sep, Me) and 32.47 (sep, Me), 36.5 (m CH_2_), 50.39, 50.44 and 50.48 (NMe), 51.26 (m CMe_2_), 69.18 (m CH).

*2,2,6,6-Tetramethyl-4-trimethylammoniopiperidine chloride hydrochloride (****3****)*. A solution of **2** monohydrate (0.98 g, 2.85 mmol) in H_2_O (7 mL) was acidified to pH 1 with conc. hydrochloric acid, and hydrogen peroxide (30%) (ca. 165 μL, 1.43 mmol) was added carefully drop-by-drop. The precipitate of crystalline iodine was filtered off and a new very small portion of hydrogen peroxide was added to verify complete oxidation of iodide. If no precipitation of iodine occurred, the solution was extracted with benzene several times to remove iodine and evaporated to dryness in vacuum to give **3** dihydrate (0.83 g, 95%), colorless crystals, mp 314-315 °C (*i*-PrOH – MeOH). Elemental analysis, found: C, 46.69; H, 10.22; N, 9.14; Cl, 23.45; calcd. for C_12_H_32_N_2_Cl_2_O_2_: C, 46.90; H, 10.50; N, 9.12; Cl, 23.07 %; IR (KBr), ν_max_ cm^−1^: 3479, 3398, 3282, 3009, 2966, 2821, 2766, 2611, 2493, 2114, 1633, 1608, 1587, 1487, 1445, 1394, 1384, 1356, 1256, 1188, 1107, 957, 903; ^1^H NMR (400 MHz, CDCl_3_ – CD_3_OD, δ): 1.41 (6H s), 1.45 (6H s), 2.04 (4H m), 3.04 (9H s), 4.24 (1H m); cf (10).

*2,2,6,6-Tetra(*^2^*H*_3_*)methyl-4-trimethylammonio(3,3,5,5-*^2^*H*_4_*, 1-*^15^*N)piperidine chloride hydrochloride (***3**-D,^15^N*)* was prepared similarly from **2**-D,^15^N. IR (KBr), ν_max_ cm^−1^: 3456, 3406, 3284, 2976, 2889, 2511, 2227, 2079, 1633, 1606, 1481, 1419, 1248, 1157, 1126, 1051, 947, 887; ^1^H NMR (300 MHz, CDCl_3_ – CD_3_OD, δ): 2.94 (9H s), 4.11 (1H m); ^13^C{^1^H} NMR (125.77 MHz, CDCl_3_, δ): 22.70 (m, Me) and 27.97 (m, Me), 33.20 (m CH_2_), 50.48 (NMe), 56.37 (m CMe_2_), 64.79 (m CH).

*2,2,6,6-Tetramethyl-4-trimethylammoniopiperidine-1-oxyl chloride (*CAT1*)*. A solution of **3** dihydrate (0.8 g, 2.6 mmol) in water (3 mL) was basified with KHCO_3_ to pH 10. Sodium tungstate (15 mg) and hydrogen peroxide (1 mL) were added to the solution and the reaction mixture was allowed to stand in dark place for 14 days. The orange solution was evaporated in vacuum to dryness, the residue was triturated with hot *i*-PrOH – MeOH mixture, the colorless inorganic precipitate was filtered off and the solvent was removed in vacuum to give CAT1 (solvate CAT1-Cl × H_2_O × (CH_3_)_2_CHOH) (0.68 g, 80%), orange crystals, mp 250 dec. (*i*-PrOH). Elemental analysis, found: C, 54.83; H, 11.08; N, 8.55; Cl 10.88; calcd. for C_12_H_26_NO_2_Cl × H_2_O × (CH_3_)_2_CHOH: C, 54.94; H, 11.07; N, 8.54; Cl, 10.81 %; IR (KBr), ν_max_cm^−1^: 3426, 3315, 3240, 3017, 2995, 2969, 1633, 1498, 1482, 1448, 1359, 1346, 1270, 1244, 1183, 1135, 1107, 975, 961, 947, 899, 670, 572.

*2,2,6,6-Tetra(*^2^*H*_3_*)methyl-4-trimethylammonio(3,3,5,5-*^2^*H*_4_*, 1-*^15^*N)piperidine-1-oxyl chloride (*^15^*N-CAT1)* was prepared similarly from 3-D,^15^N. IR (KBr), ν_max_ cm^−1^: 3425, 3242, 3018, 2966, 2245, 2229, 2125, 2075, 1500, 1479, 1383, 1344, 1184, 1136, 1049, 958, 893, 821, 617, 513.

*1-Hydroxy-2,2,6,6-tetramethyl-4-trimethylammoniopiperidine chloride (*^14^*N*-CAT1H*)*. The nitroxide CAT1 (0.65 g, 2 mmol) was dissolved in a mixture of isopropanol (1 mL), ethanol (1 mL) and conc. hydrochloric acid (1 mL). The mixture was stirred overnight and dehydrated in vacuum to dryness. The residue was crystallized from a mixture *i*-PrOH – MeOH 10:1 to give CAT1H (solvate CAT1H-Cl × (CH_3_)_2_CHOH) (0.55 g, 80%), mp 270-273°C. Elemental analysis, found: C, 51.80; H, 10.35; N, 8.29; Cl, 20.16; calcd. for C_12_H_28_N_2_OCl_2_ × (CH_3_)_2_CHOH: C, 54.94; H, 11.07; N, 8.54; Cl, 10.81%; IR (KBr), ν_max_ cm^−1^: 1643, 1479, 1401, 1392, 1355, 1261, 1244, 1138, 1105, 1065, 955, 908, 855; ^1^H NMR (300 MHz, CD_3_OD, δ): 1.58 (6H s), 1.68 (6H s), 2.54 (4H m), 3.26 (9H s), 4.29 (1H m), *i*-PrOH: 1.18 (6H d, J 6 Hz), 3.95 (1H sep., J 6 Hz); ^13^C{^1^H} NMR (75 MHz, CD_3_OD, δ): 20.35 and 28.17 (Me), 36.63 (CH_2_), 52.10 (N-CH_3_), 65.41 (CH), 69.38 (CMe_2_), cf (10).

*1-Hydroxy-2,2,6,6-tetra(*^2^*H*_3_*)methyl-4-trimethylammonio(3,3,5,5-*^2^*H*_4_*,1-*^15^*N)piperidine chloride (*^15^*N-*CAT1H*)* was prepare similarly from ^15^*N*-CAT1. IR (KBr), ν_max_ cm^−1^: 3261, 3020, 2969, 2915, 2723, 2486, 2375, 2231, 2046, 1583, 1489, 1471, 1377, 1298, 1244, 1159, 1132, 1051, 955, 887, 820; ^1^H NMR (400 MHz, CD_3_OD, δ): 3.25 (9H s), 4.21 (1H m), *i*-PrOH: 1.18 (6H d, J 6 Hz), 3.95 (1H sep., J 6 Hz).

*1-Hydroxy-2,2,6,6-tetramethyl-4-[2-(triphenylphosphonio)acetamido]piperidinium dichloride (mitoTEMPO-H)* was prepared via catalytic hydrogenation of mitoTEMPO on Pd/C in analogy to previously described procedure (12) and isolated as trihydrate *(mitoTEMPO-H*×*HCl*×*3 H*_2_*O)*. Yield 80%, colorless crystals, mp 188-191°C (reprecipitated from isopropanol with diethyl ether). ^1^H NMR (CDCl_3_) δ= 1.22 (6H, s, 2 × CH_3_ axial), 1.42 (6H, s, 2 × CH_3_ equatorial), 1.86 (2H, d J = 13 Hz, 2 × CH axial, CH_2_), 2.00 (2H, dd J_1_ = 13 Hz, J_2_ = Hz, 2 × CH equatorial, CH_2_), 3.85 (1H, m, CH-N), 4.94 (2H, d J_P_ = 14 Hz, PCH_2_), 7.59 (6H, m, o-Ph), 7.72 (9H, m, m,p-Ph), 9.54 (1H br. s, NH), 11.09 (1H, s OH). ^13^C NMR (CDCl_3_) 20.4, 27.9 (CH_3_); 32.3 (d J_P_ = 55 Hz, PCH_2_), 40.5 (CH), 40.7 (CH_2_); 68.1 (CMe_2_), 118.1 (d J_P_ = 88 Hz, C_i_, Ph), 130.2 (d J_P_ = 26 Hz, C_o_, Ph), 133.9 (d J_P_ = 10 Hz, C_m_, Ph), 134.9 (d J_P_ = 2 Hz, C_p_, Ph), 162.4 (d J_P_ = 0.5 Hz, C=O). IR (KBr) 1668 (C=O), 1553, 1439, 1388, 1335, 1112, 996, 746, 720, 690, 516. Anal. Found: C, 57.81; H, 6.95; N, 4.66; Cl, 11.50; P, 5.38. Calcd. For C_29_H_37_Cl_2_N_2_O_2_P ×3 H_2_O: C, 57.90; H, 7.21; N, 4.66; Cl, 11.79; P, 5.15.

### Animals

Wild-type C57BL/6J and mice lacking the gp91 phox catalytic subunit of phagocytic NADPH oxidase (Nox2KO) were obtained from Jackson Laboratories. Some animals were infused with low-suppressor dose of angiotensin II (0.3 mg/kg/day) for 14 days as described previously (11). BP was monitored by the tail cuff method as described previously (13). Vanderbilt Institutional Animal Care and Use Committee approved all procedures. Sham and angiotensin II infused C57Bl/6J and Nox2KO mice were sacrificed by carbon dioxide and spleens were immediately removed and placed in the ice-cold isolation medium for splenocyte isolation (5).

### Cellular experiments

In initial experiments splenocytes were treated with either vehicle (saline), TNFα (1 ng/ml) or IL-17A (10 ng/ml) for 3-h at 37C. To induce superoxide production as a “positive control,” unstimulated splenocytes were treated acutely with NADPH oxidase activator PMA (10 μM) (8) or opener of K_ATP_ channel diazoxide (0.1 μM) specifically inducing mitochondrial superoxide (14). To investigate the potential between mitochondrial superoxide and phagocytic NADPH oxidase *in vivo*, splenocytes (10^7^/ml) isolated from Sham and angiotensin II infused C57Bl/6J and Nox2KO mice were treated acutely with antimycin A, CCCP, PMA, rotenone or Cu,Zn-SOD and then incubated with spin probes.

### EPR analysis

In cell-free studies, spin probes were incubated with xanthine (0.2 mM) and xanthine oxidase (2 mUnits/ml) superoxide generating system. Specificity of superoxide detection was confirmed by blocking the reaction with Cu,Zn-SOD (50 U.ml). In cellular experiments, splenocytes (10^7^/ml) were incubated with ^15^N-CAT1H (500 μM) and/or ^14^N-mitoTEMPO-H (25 μM) for 30 min at 37°C prior to EPR analysis. Extracellular O_2_^•^ was measured by accumulation of ^15^N-CAT1 nitroxide following low field component of EPR spectra. Mitochondrial O_2_^•^ production was measured by accumulation of ^14^N-mitoTEMPO nitroxide following central field component of EPR spectra. The potential contamination of ^14^N-mitoTEMPO signal with extracellular superoxide did not exceed 5%, since the amount of ^14^N-mitoTEMPO-H is 20-fold lower than ^15^N-CAT1H spin probe (25 μM and 500 μM accordingly), and these probes have similar reaction rate constant with superoxide. This was confirmed experimentally in the xanthine oxidase superoxide system containing ^14^N-mitoTEMPO-H (25 μM) and ^15^N-CAT1H (500 μM) spin probes (Supplementary material 1). Experimental ratio of ^14^N-mitoTEMPO and ^15^N-CAT1 EPR signals in cellular samples was in the range between 32 and 54%, therefore, contamination of ^14^N-mitoTEMPO signal with extracellular superoxide was negligible.

EPR measurements were performed in 50 μL glass capillary tubes at room temperature using the Bruker EMX spectrometer. Spectrometer settings were as follows: field sweep, 60 G; microwave frequency, 9.82 GHz; microwave power, 20 mW; modulation amplitude, 1 G; conversion time, 164 ms; time constant 328 ms; sweep time, 168 s; receiver gain, 1 ×10^5^; number of scans, 4. The rate of O_2_^•^ formation was measured by monitoring the amplitude of the low-field component of the EPR spectrum as previously described (7). The concentration of nitroxide was determined from a calibration curve for intensity of the EPR signal of TEMPOL at various known concentrations. The rate of O_2_^•^ production was calculated from the accumulation of nitroxide, obtained from the EPR time scan. For this purpose, the EPR kinetics were analyzed using linear regression and WinEPR software (BrukerBiospin Corp, Billerica, MA).

### Statistics

Experiments were analyzed using the Student Neuman Keuls *post-hoc* test and the analysis of variance (ANOVA). *P* levels < 0.05 were considered significant.

## Results

### Simultaneous EPR detection of superoxide by ^15^N- and ^14^N- hydroxylamine spin probes in xanthine oxidase system

**Figure 2:**
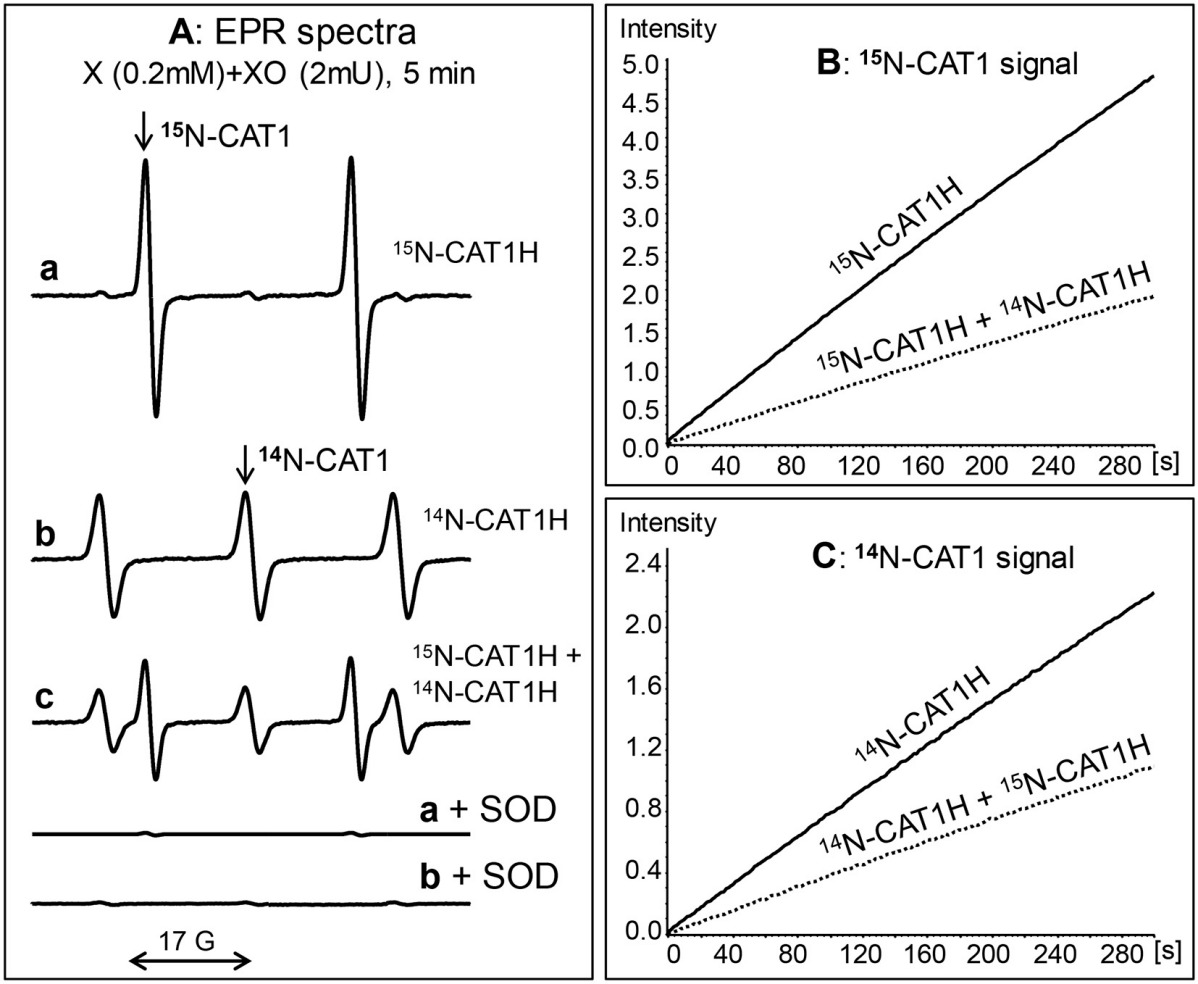
Simultaneous detection of superoxide by ^15^N- and ^14^N- hydroxylamine spin probes. **(A)** EPR spectra of hydroxylamine spin probes incubated with xanthine (0.2 mM) and xanthine oxidase (2 mUnits/ml) for 5 min: 0.2 mM ^15^N-CAT1H (a), 0.2 mM CAT1H (b), and 0.2 mM ^15^N-CAT1H plus 0.2 mM ^14^N-CAT1H (c). Accumulation of both ^15^N-CAT1 and ^14^N-CAT1 was inhibited in the presence of superoxide dismutase (SOD, 50 units/ml). **(B)** EPR intensity of ^15^N-CAT1 in the sample with xanthine oxidase superoxide generating system and ^15^N-CAT1H or ^15^N-CAT1H+^14^N-CAT1H. **(C)** ESR intensity of ^14^N-CAT1 in the sample with xanthine oxidase superoxide generating system and ^14^N-CAT1H or ^14^N-CAT1H+^14^N-CAT1H. Figure shows typical ESR spectra of three independent experiments.

Superoxide detection by ^15^N-CAT1H spin probe was studied in xanthine oxidase superoxide generating system using field scan and time scan modes. Incubation of 0.2 mM ^15^N-CAT1H with xanthine oxidase and xanthine leads to robust accumulation of ^15^N-CAT1H nitroxide as measured by appearance of doublet EPR spectra (^15^N-hyperfine splitting constant a_N_ = 23.5 G) (Figure 2A, a, a+SOD). The superoxide detection can be also followed by the time scan of low field component ^15^N-CAT1H nitroxide (Figure 2B). Superoxide detection with ^15^N-CAT1H spin probe was compared with conventional ^14^N-containing CAT1H. Incubation of ^14^N-CAT1H spin probe with xanthine oxidase system yielded ^14^N-CAT1 nitroxide (a_N_ = 16.7 G). Interestingly, intensity of EPR signal of ^15^N-CAT1H nitroxide was about 2-fold higher compared with CAT1 due to distribution of EPR intensity into 2-line signal vs. 3-line and narrowing of CAT1 signal due to replacement of hydrogen to deuterium (Figures 2A–C). Supplementation of 50 units/mL of Cu,Zn-superoxide dismutase completely inhibited the formation of ^15^N- and ^14^N-containing CAT1 nitroxide products. To test the possibility of simultaneous detection of superoxide by ^15^N- and ^14^N-containing spin probes we performed EPR studies using mixture ^15^N-CAT1H and ^14^N-CAT1H in xanthine oxidase superoxide generating system. Analysis of the field scan showed the presence of both ^15^N- (a_N_ = 23.5 G) and ^14^N-containing CAT1 (a_N_ = 16.7 G) nitroxides in equimolar ratio. EPR spectra of ^15^N- and ^14^N-containing CAT1 nitroxides had a very good separation allowing to track accumulation of these nitroxides independently (Figures 2B,C).

### Simultaneous EPR detection of extracellular and mitochondrial O_2_^•^ by ^15^N-CAT1H and ^14^N-mitoTEMPO-H in splenocytes

**Figure 3:**
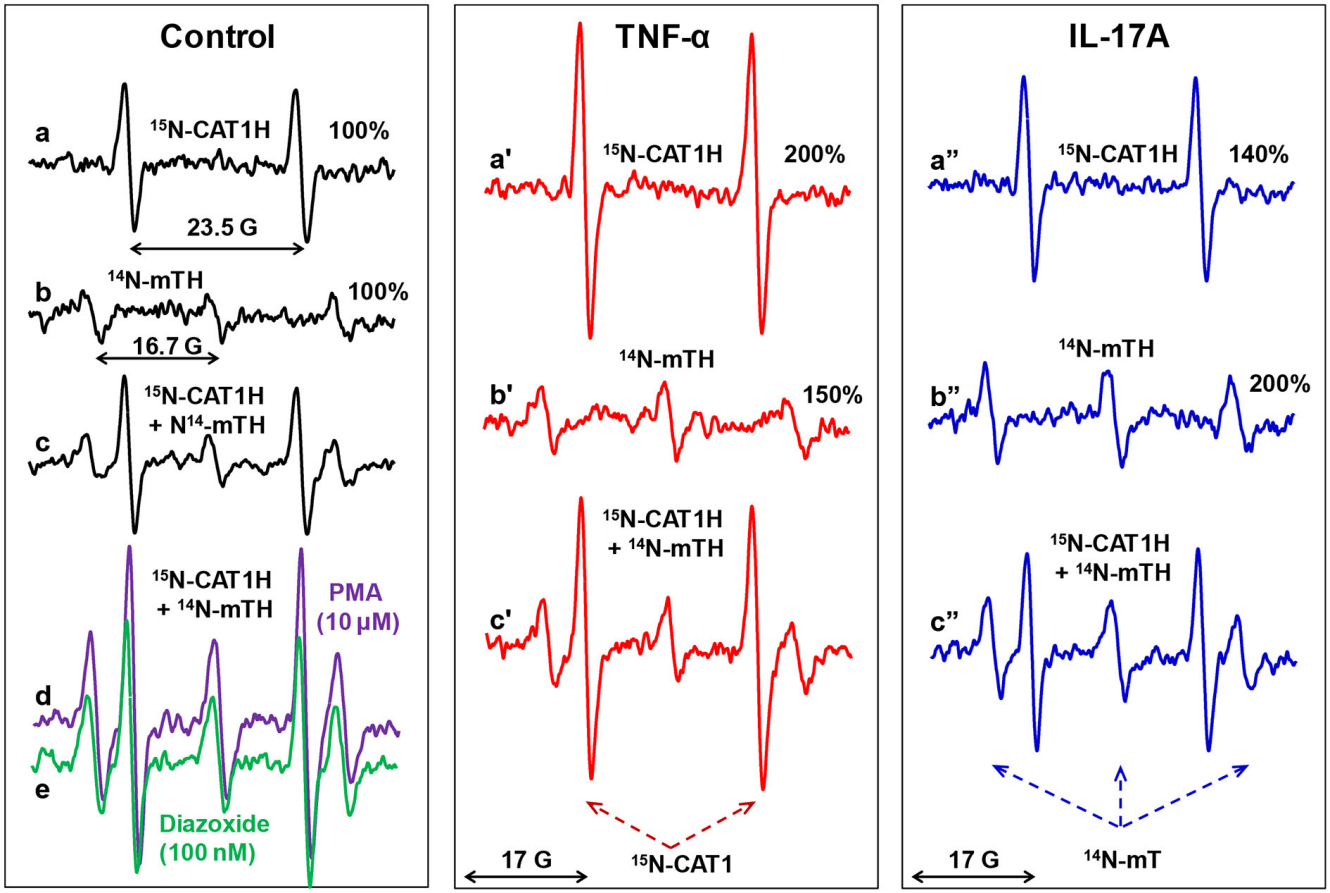
Simultaneous EPR detection of extracellular and mitochondrial O_2_^•^ by ^15^N-CAT1H and ^14^N-mitoTEMPO-H (^14^N-mTH) spin probes in splenocytes. Mouse splenocytes were stimulated *ex vivo* with TNFα (1 ng/ml) or IL-17A (10 ng/ml) for 3-h. Cells (10^7^/ml) were supplemented with ^14^N-CAT1H (0.5 mM) and/or ^14^N-mitoTEMPO-H (25 μM) and incubated for 30 min at 37 °C prior to EPR analysis. Unstimulated splenocytes were treated acutely with NADPH oxidase activator PMA (10 μM) or inducer of mitochondrial superoxide diazoxide (0.1 μM) as a “positive control” for increased superoxide production. Figure shows typical ESR spectra of four independent experiments.

Validation of site-specific superoxide detection in splenocytes was performed by analysis of extracellular superoxide with ^15^N-CAT1H or mitochondrial O_2_^•^ by ^14^N-mitoTEMPO-H. Then we tested simultaneous detection of extracellular and mitochondrial O_2_^•^ in the presence of mixture of ^15^N-CAT1H and ^14^N-mitoTEMPO-H. Incubation of control splenocytes with ^15^N-CAT1H or ^14^N-mitoTEMPO-H produced detectable amounts of corresponding nitroxides. Interestingly, the amount of ^15^N-CAT1 and ^14^N-mitoTEMPO accumulated in splenocytes upon incubation with a mixture of ^15^N-CAT1H and ^14^N-mitoTEMPO-H was identical to the amount of these nitroxides in the samples containing only one spin probe (Figure 3, traces a–c) indicating that simultaneous presence of both hydroxylamines and both nitroxides does not affect individual results of site-specific superoxide detection of extracellular and mitochondrial O_2_^•^.

We treated splenocytes with a known NADPH oxidase activator PMA (10 μM) or inducer of mitochondrial superoxide diazoxide (0.1 μM) as a “positive control” for increased superoxide production. Interestingly, PMA increased not only extracellular superoxide production by phagocytic NADPH oxidase as measured by increased ^15^N-CAT1H doublet signal but also increased mitochondrial triplet signal of ^14^N-mitoTEMPO (Figure 3, trace d). Paradoxically, inducer of mitochondrial superoxide diazoxide did increase the phagocytic NADPH oxidase activity (^15^N-CAT1H doublet signal) as well as mitochondrial superoxide (triplet signal of ^14^N-mitoTEMPO) (Figure 3, trace e). The potential interplay between extracellular and mitochondrial O_2_^•^ was further investigated in splenocyte stimulated with pro-inflammatory cytokines TNFα and IL-17A. TNFα increased extracellular O_2_^•^ by 200% and mitochondrial O_2_^•^ by 150%. Simultaneous extracellular and mitochondrial O_2_^•^ by mixture of ^15^N-CAT1H and mitoTEMPO-H confirmed these results (Figure 3, traces a'–c'). Analysis of IL-17A stimulated splenocytes showed increased extracellular O_2_^•^ by 140% and mitochondrial O_2_^•^ by 200% confirmed by simultaneous use of ^15^N-CAT1H and ^14^N-mitoTEMPO-H (Figure 3, traces a”–c”). Although cytokines showed distinct effects on phagocytic NADPH oxidase activity and mitochondrial O_2_^•^, there is a pattern of co-stimulation of both extracellular and mitochondrial O_2_^•^ suggesting coupling between phagocytic NADPH oxidase and mitochondria.

### EPR analysis of extracellular and mitochondrial O_2_^•^ in splenocytes from wild-type and Nox2KO mice

The interplay between mitochondrial superoxide and activity of phagocytic NADPH oxidase was further investigated in splenocytes isolated from Sham and angiotensin II infused C57Bl/6J and Nox2KO mice. Angiotensin II is known to stimulate NADPH oxidase activity via AT1 receptor. Sub-pressor dose of angiotensin II does not significantly raise blood pressure in wild-type mice (15) but promotes the inflammatory cell activation (16). We used low dose of angiotensin II infusion to stimulate the splenic cells *in vivo*. Following 14-days of angiotensin II infusion (0.3 mg/kg/day) mice were sacrificed and spleens were isolated for splenocytes preparation.

**Figure 4:**
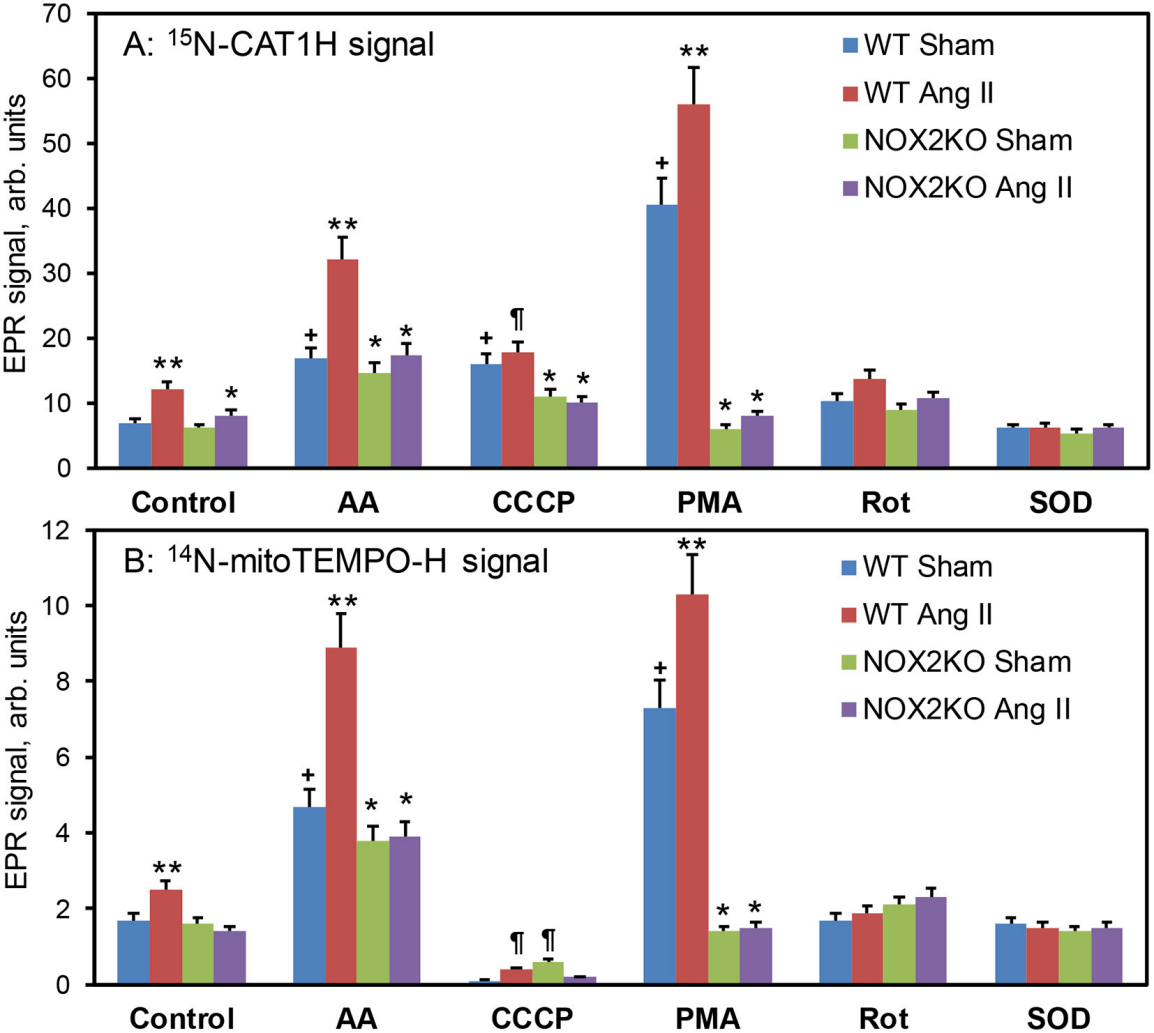
EPR analysis of extracellular and mitochondrial O_2_^•^ by ^15^N-CAT1H **(A)** and ^14^N-mitoTEMPO-H **(B)** spin probes in splenocytes. Wild-type C57Bl/6J and Nox2KO mice were infused with saline (Sham) or angiotensin II (Ang II, 0.3 mg/kg/day) for 14 days prior to EPR studies. Isolated splenocytes were placed in Krebs-Hepes buffer (10^7^/ml) and treated acutely with antimycin A, CCCP, PMA, rotenone or Cu,Zn-SOD and incubated with ^15^N-CAT1H (500 μM) and ^14^N-mitoTEMPO-H (25 μM) for 30 min at 37 °C prior to EPR analysis. Extracellular O_2_^•^ production was measured by accumulation of ^15^N-CAT1H nitroxide following low field component of EPR spectra. Mitochondrial O_2_^•^ production was measured by accumulation of ^14^N-mitoTEMPO nitroxide following central field component of EPR spectra. Results are mean ± SEM (*n* = 6). *P < 0.01 vs. WT+Ang II, **P < 0.05 vs WT Sham, ^¶^*P* < 0.01 vs. Control, ^**+**^*P* < 0.01 vs. WT Sham.

Angiotensin II infusion in wild-type mice increased the extracellular splenocyte superoxide (^15^N-CAT1H signal) which was further enhanced by complex III inhibitor antimycin A, mitochondrial uncoupling agent CCCP and NADPH oxidase agonist PMA (Figure 4A). Nox2 depletion attenuated angiotensin II mediated stimulation and inhibited both extracellular and mitochondrial PMA-induced superoxide production. Interestingly, EPR analysis of mitochondrial superoxide production (^14^N-mitoTEMPO-H signal) showed substantial increase in wild-type splenocytes but not in Nox2KO (Figure 4B). Addition of antimycin A increased mitochondrial superoxide in all splenocyte groups particularly in splenocytes isolated from angiotensin II-infused wild-type mice. In contrast to ^15^N-CAT1H signal, addition of CCCP inhibited ^14^N-mitoTEMPO-H signal which may be associated with reduction of mitochondrial potential in CCCP treated cells which can reduce both mitochondrial superoxide production and accumulation of mitoTEMPO-H spin probe in mitochondrial matrix. Acute PMA treatment increased mitochondrial superoxide in wild-type splenocytes but not in Nox2KO cells supporting the induction of mitochondrial superoxide by Nox2-dependent mechanism. These data indicate that splenocytes isolated from hypertensive angiotensin II-infused mice are “primed” for enhanced superoxide production from both phagocytic NADPH oxidase and mitochondria. Furthermore, Nox2 depletion attenuated splenic superoxide production not only by phagocytic NADPH oxidase and but also by mitochondria (Figures 4A,B).

## Discussion

Our data demonstrate that ^15^N-and deuterium-enriched hydroxylamine spin probe ^15^N-CAT1H provides high sensitivity superoxide measurements and combination with ^14^N-mitoTEMPO-H allows simultaneous detection of extracellular and mitochondrial superoxide. We found that EPR spectra of ^15^N- and ^14^N-containing nitroxides had a very good separation allowing independent measurements of these nitroxides. Simultaneous presence of both hydroxylamines and both nitroxides does not affect individual results of site-specific superoxide detection of extracellular and mitochondrial O_2_^•^. Detection of extracellular superoxide production by phagocytic NADPH oxidase was validated by positive control with PMA, inhibition of EPR signal by NOX2 depletion or supplementation with extracellular superoxide dismutase. Detection of mitochondrial superoxide was validated by inducers of mitochondrial superoxide diazoxide and antimycin A, inhibitor of mitochondrial superoxide CCCP.^17^ We suggest that these probes can be used to study the site-specific superoxide production and analysis of important sources of oxidative stress in cardiovascular conditions.

**Figure 5:**
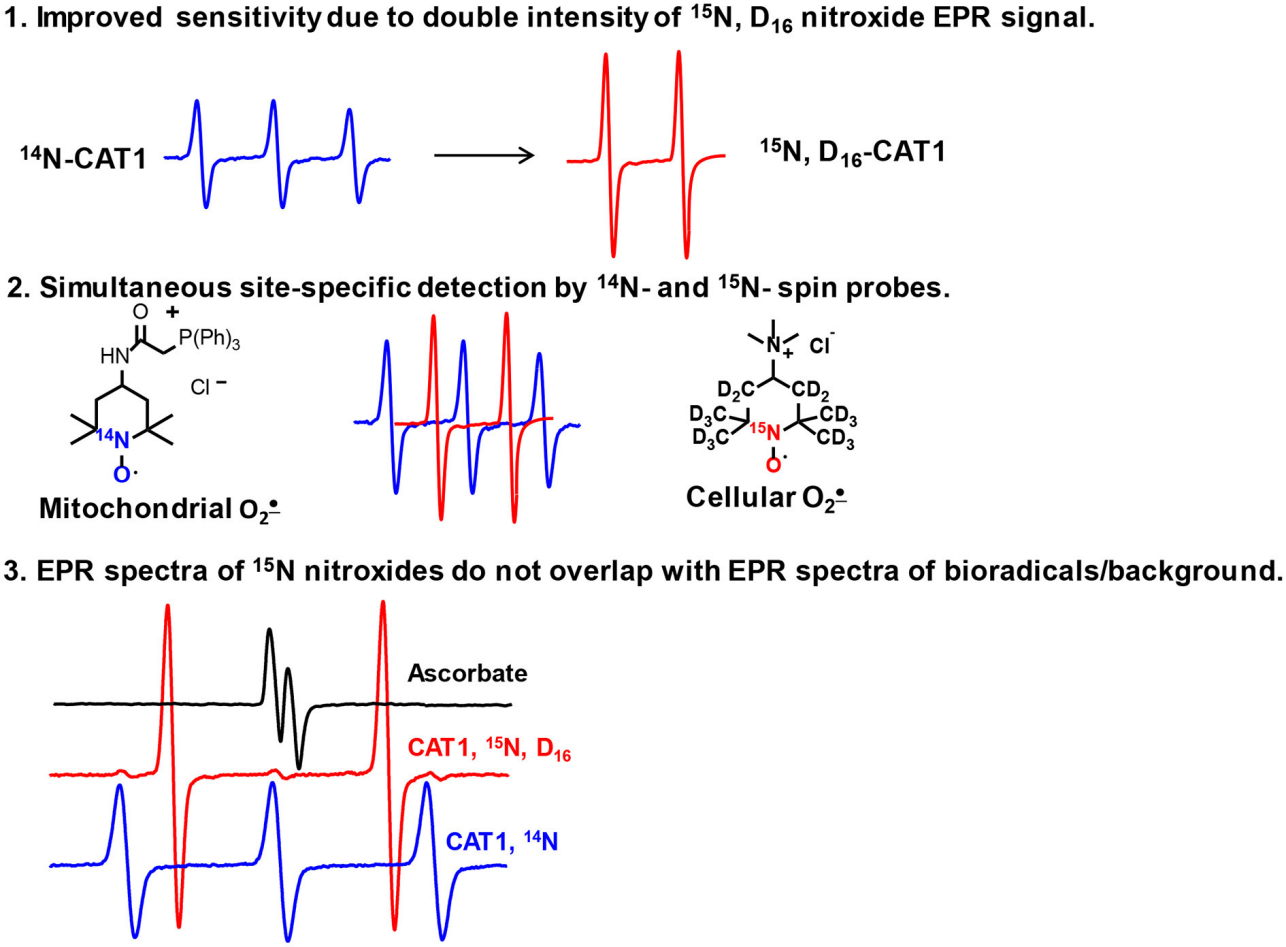
Advantages of ^15^N- and deuterium-enriched hydroxylamine spin probes.

The advantages of ^15^N-labeled nitroxides over their conventional ^14^N analogs result from the properties of nuclei of these stable nitrogen isotopes. In contrast to conventional nitroxides with ^14^N-isotope, which gives a triplet EPR spectrum due to nitrogen nuclear spin S = 1, the ^15^N with S = 1/2 gives doublet spectrum. The lower number of transitions gives more intense spectral lines due to distribution of EPR signal in two-line (^15^N) vs. three lines (^14^N). Replacement of ^1^H to ^2^H (deuterium) decreases the observed linewidth because of much smaller hyperfine-coupling constant on deuterium nuclei compared with protons which results in EPR line narrowing leading to further increase in the intensity of EPR spectral lines. The utility of simultaneous use of ^15^N- and ^14^N-labeled nitroxides in cellular/tissue distribution studies have been shown previously, (17) however this approach have never been used for hydroxylamine spin probes and superoxide detection in different cellular compartments. Our work reveals several advantages of ^15^N-and deuterium-enriched hydroxylamine spin probes, namely, i) improved sensitivity in superoxide detection; ii) independent detection of ^15^N- and ^14^N- spin probes in distinct cellular compartments; iii) EPR signal of ^15^N-spin probe does not overlap with spectra of biological radicals and paramagnetic background (Figure 5).

We have previously shown that phagocytic NADPH oxidase predominantly produces extracellular superoxide (18). Interestingly, mitochondrial alterations increase the activity of phagocytic NADPH oxidase via redox and Ca^2+^-dependent pathways (19, 20). In endothelial cells, activation of Nox2 promotes production of mitochondrial superoxide which can be attenuated by Nox2 depletion or Nox2 inhibition (21). Meanwhile, we did not know the time-dependence of the mitochondria-Nox2 crosstalk. Typically, cellular regulation occurs consequentially, one after another, therefore, increase in one signal translates later in the stimulation of secondary signal. In this work we studied the production of extracellular and mitochondrial superoxide in splenic cells. The relative and absolute intensities of site-specific superoxide production depend on particular stimulation such as IL-17A, TNFα, or PMA. Meanwhile, our measurements revealed the parallel increase of both extracellular and mitochondrial superoxide in all models suggesting a tight association between phagocytic NADPH oxidase activity and mitochondrial superoxide production. This mechanism can promote inflammatory activation in metabolic conditions associated with mitochondrial impairment and increased mitochondrial superoxide. On the other hand, inflammation can trigger metabolic/mitochondrial dysfunction due to the same Nox2-mitochondrial crosstalk in immune cells.

We have previously reported a robust crosstalk between Nox2 activity and mitochondrial superoxide production in endothelial cells (21, 22). Interestingly, targeted inhibition of Nox2 or mitochondria-targeted antioxidants were able break this vicious cycle and downregulate cellular superoxide production and rescue endothelial function. For example, treatment of hypertensive mice with malate and mitoTEMPO reduced oxidative stress, improved endothelial function and diminished hypertension (21). The role of inflammation and immunity in hypertension has been recently described (23). It was found that phagocytic NADPH oxidase in immune cells plays a critical role in the development of hypertension (4). Interestingly, treatment of T cells isolated from hypertensive mice with mitochondria-targeted superoxide scavenger mitoTEMPO significantly abrogated the pro-hypertensive response of immune cells (24). These data support the Nox2-mitoxhondria interplay both in non-phagocytic and immune cells. Furthermore, this work suggests very quick and robust Nox2-mitochondrial crosstalk which can be potentially targeted for treatment of cardiovascular conditions.

It must be noted that interplay between mitochondrial superoxide and NADPH oxidases is not limited to Nox2. On one hand, mitochondrial oxidants promote redox and calcium dependent activation of Nox1 and Nox5 in non-phagocytic cells. On the other hand, increased activity of Nox4 (producing intracellular ROS) can increase mitochondrial superoxide production via H_2_O_2_ and p66Shc dependent mechanisms (25). The pathophysiological role of mitochondrial interactions with specific NADPH isoforms warrants additional studies.

## Conclusion

Inflammation and oxidative stress play an important role in pathogenesis of cardiovascular disease. Development of new treatments targeting these pathways could improve the treatment of many pathological conditions. Blocking inflammatory pathways is hindered by the off-target effects on the immune system and non-discriminatory antioxidant treatment can impair redox cell signaling pathways. Understanding the precise regulations of immune cells and oxidant production can help in the development of new therapies targeting specific inflammatory and oxidative stress mechanisms. In this work we described the interplay between phagocytic NADPH oxidase activity and mitochondrial superoxide production in immune cells stimulated by pro-inflammatory cytokines, mitochondrial agents or angiotensin II. This crosstalk between NADPH oxidase and mitochondria in inflammatory cell can promote inflammatory injury and end-organ-damage. Indeed, metabolic conditions are associated with both mitochondrial dysfunction and upregulation of pro-inflammatory pathways. We suggest that specific targeting Nox2 or mitochondrial function can break this pathophysiological vicious cycle (3), reduce inflammation and improve end-organ function.

## Data availability statement

The original contributions presented in the study are included in the article/Supplementary material, further inquiries can be directed to the corresponding author.

## Ethics statement

The animal study was reviewed and approved by Vanderbilt Institutional Animal Care and Use Committee.

## Author contributions

SD conceived, designed research, drafted manuscript, and edited and revised manuscript. AD performed animal and cellular studies and analyzed data. IK synthesized the spin probes. AD and IK interpreted results of experiments and approved final version of manuscript. All authors contributed to the article and approved the submitted version.

## Funding

This work was supported by funding from National Institutes of Health (R01HL144943 and RO1HL157583) and American Heart Association Transformational Project Award (19TPA34910157).

## Conflict of interest

The authors declare that the research was conducted in the absence of any commercial or financial relationships that could be construed as a potential conflict of interest.

## Publisher's note

All claims expressed in this article are solely those of the authors and do not necessarily represent those of their affiliated organizations, or those of the publisher, the editors and the reviewers. Any product that may be evaluated in this article, or claim that may be made by its manufacturer, is not guaranteed or endorsed by the publisher.
