# Supplementary material for: Coupling of phagocytic NADPH oxidase activity and mitochondrial superoxide production
Source: Front Cardiovasc Med. 2022 Jul 28;9:942736. doi: 10.3389/fcvm.2022.942736 (PMC9366351; doi:10.3389/fcvm.2022.942736)
Supplement: Supplementary file 1 [file Data_Sheet_1.PDF]

Supplement 1.

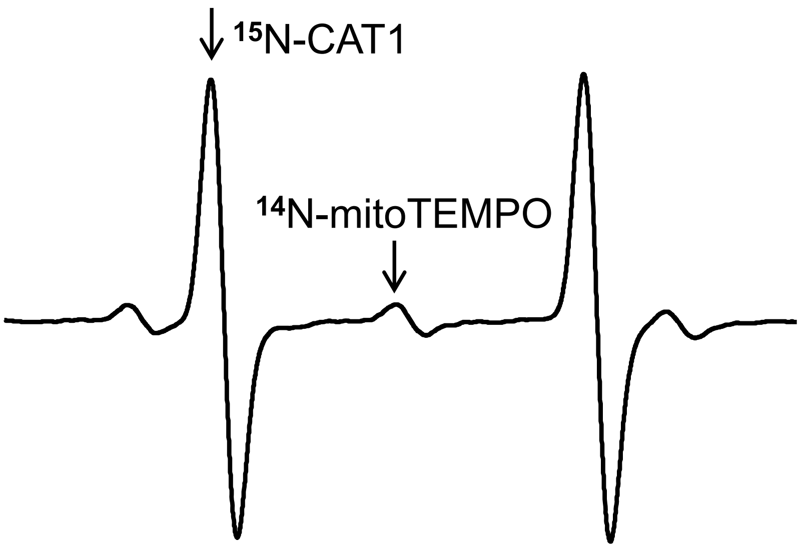

EPR spectra of ^{15}N-CAT1H (500μM) and ^{14}N-mitoTEMPO-H (25μM) spin probes in Xanthine (0.2 mM) plus Xanthine Oxidase (2 mUnits/ml) system.
